# Multi-colorized tint map for distinguishing triple-negative breast cancers from cysts and fibroadenomas based on the tumor margin

**DOI:** 10.3389/fonc.2026.1741453

**Published:** 2026-02-05

**Authors:** Hongjin Xiang, Xiaoyu Chen, Xiao Tan, Xiangmei Chen, Heng Lv, Desheng Sun

**Affiliations:** Department of Ultrasound, Peking University Shenzhen Hospital, Shenzhen Peking University-The Hong Kong University of Science and Technology Medical Center, Shenzhen, Guangdong, Shenzhen, Guangdong, China

**Keywords:** HSV color space, multi-colorized tint maps, peripheral halo sign, triple-negative breast cancer, ultrasound

## Abstract

**Background:**

Triple-negative breast cancer (TNBC) can appear markedly hypoechoic on grayscale ultrasound and may mimic benign entities such as cysts and fibroadenomas, complicating preoperative differentiation. Because the human visual system is more sensitive to multi-color patterns than to subtle grayscale contrast, we investigated whether hue, saturation, and value (HSV)-based multi-colorized tint maps could improve visual discrimination of TNBC from benign, extremely hypoechoic breast lesions.

**Methods:**

This single-center retrospective study included 48 histopathologically confirmed, extremely hypoechoic lesions (16 TNBCs, 16 cysts, and 16 fibroadenomas). Grayscale ultrasound images were post-processed into HSV tint maps using two pre-specified color-coding strategies. A single intensity threshold (135) was predefined and then fixed across all cases to enhance reproducibility; its stability was examined in sensitivity analyses around the selected value. Two radiologists independently reviewed grayscale and colorized images in separate, blinded sessions with randomized case order and a washout interval between sessions to reduce recall bias. Diagnostic performance and inter-reader agreement (Cohen’s κ) were assessed.

**Results:**

Multi-colorized tint maps enhanced subtle marginal echogenic transitions. TNBC more frequently exhibited a distinctive peripheral “halo” pattern, whereas this feature was uncommon in cysts and fibroadenomas. For differentiating TNBC from benign lesions, sensitivity/specificity improved from 75%/69% on grayscale to 88%/91% with multi-colorization. Inter-reader agreement for the colorized interpretation was high (κ =0.88).

**Conclusion:**

HSV-based multi-colorized tint maps reveal a visually intuitive marginal “halo” feature that may assist preoperative differentiation of TNBC from benign, extremely hypoechoic lesions and can improve diagnostic performance compared with grayscale ultrasound. Future multicenter studies should validate robustness across ultrasound vendors and prospectively correlate the halo with histopathologic characteristics (e.g., cellularity and stromal response).

## Introduction

Triple-negative breast cancer (TNBC) is a biologically aggressive subtype defined by the absence of estrogen receptor (ER), progesterone receptor (PR), and HER2 expression, which limits the utility of endocrine therapy and HER2-targeted regimens such as trastuzumab (1). TNBC carries a higher risk of early relapse and poorer survival than other molecular subtypes, with a reported 5-year relative survival around the high 70% range (2). It accounts for approximately 10%–20% of breast cancers and is more prevalent in younger women (1, 3). In clinical practice, earlier recognition is consequential: prompt diagnosis can accelerate neoadjuvant chemotherapy and increase the likelihood of achieving a pathologic complete response (pCR), which may support surgical de-escalation and broaden eligibility for breast-conserving surgery (BCS) (4–6).

Despite this need, imaging-based differentiation of TNBC remains challenging. While many malignancies present as irregular masses with spiculated margins, TNBC may appear round or oval and can be misclassified across modalities (7, 8). Ultrasound (US), given its accessibility and routine use in evaluating breast masses, is frequently relied upon for first-line assessment. However, TNBC can exhibit circumscribed margins, parallel orientation, markedly hypoechoic to near-anechoic echogenicity, and posterior acoustic enhancement, thereby resembling complicated cysts or fibroadenomas on conventional grayscale US (8). This diagnostic overlap is often driven by subtle boundary transitions at the lesion margin—visual information that may be present in B-mode data yet insufficiently salient for consistent human interpretation in time-pressured routine reading.

Recent research in breast imaging has therefore moved beyond conventional grayscale appearance to explore strategies that enrich lesion contrast and interpretability. Emerging modalities and computer-aided approaches, including hyperspectral imaging and related multi-/spectral techniques, have been actively investigated for improving breast cancer detection and characterization (9–11). For example, a recent systematic meta-analysis of computer-aided detection using hyperspectral imaging reported encouraging diagnostic performance across studies, highlighting the potential value of expanding information beyond standard grayscale intensity (9). Likewise, contemporary reviews summarize a growing pipeline of innovative imaging techniques and their translational barriers, underscoring that many promising approaches still depend on additional hardware, specialized acquisition protocols, or computational infrastructure that can impede adoption in typical ultrasound workflows (10). At the same time, broader breast cancer research continues to emphasize real-world constraints—ranging from patient-level determinants to health-system variability—that shape the feasibility and uptake of new diagnostic tools (11). Collectively, this landscape motivates methods that are not only technically effective but also operationally lightweight and easily integrated into existing clinical pipelines.

A practical, workflow-compatible alternative is to improve the perceptual salience of information already contained in standard B-mode images through transparent and reproducible visualization. Human visual perception is generally more sensitive to color differences than to fine grayscale gradients (12). In color science, hue, saturation, and value (HSV) is a perceptually intuitive color space: hue encodes color type, saturation encodes color vividness, and value encodes brightness (13). In ultrasonography, tint maps (pseudo-color maps) transform grayscale images into color displays and can help highlight tissue transitions or boundaries (14). Conventional tint maps often apply a single hue scale; by contrast, multi-hue schemes may amplify perceptual separation and make gradual boundary changes more conspicuous without altering the underlying B-mode signal (14, 15). However, evidence remains limited regarding whether such multi-color visualization can systematically improve preoperative discrimination of TNBC from benign, extremely hypoechoic lesions under routine visual interpretation conditions (16, 17).

Therefore, this study evaluated an HSV-based, multi-colorized tint-map post-processing strategy designed to emphasize marginal echogenic transitions on standard grayscale US images. We tested whether multi-colorization improves the visual identification of a peripheral “halo”-like pattern at lesion margins and whether this translates into improved diagnostic performance compared with grayscale interpretation when distinguishing TNBC from cysts and fibroadenomas.

## Methods

### Study design and patients

This single-center retrospective study was approved by the Ethics Committee of Peking University Shenzhen Hospital, with a waiver of written informed consent due to the retrospective use of anonymized imaging data (as applicable). Consecutive patients who underwent breast ultrasound for suspicious breast lesions between January 2022 and June 2024 were screened.

Because TNBC may mimic benign, extremely hypoechoic lesions on ultrasound, a balanced dataset of 48 lesions with comparable grayscale appearances was assembled: 16 TNBCs, 16 cysts, and 16 fibroadenomas. TNBCs and fibroadenomas were confirmed by histopathology, whereas cysts were confirmed by aspiration findings. For each lesion, the representative B-mode image of the primary lesion acquired before biopsy, surgery, neoadjuvant chemotherapy, or any other intervention was extracted for post-processing and analysis. Images with substantial artifacts or incomplete lesion depiction were excluded.

### Image processing

**Figure 1:**
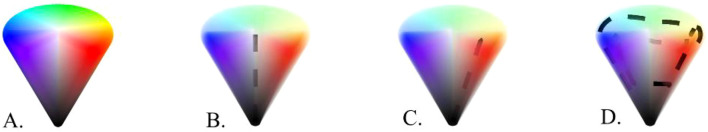
**(A)** Schematic of the HSV color space. **(B–D)** Trajectories used for **(B)** grayscale, **(C)** single-colorized tint mapping, and **(D)** multi-colorized tint mapping. HSV, hue, saturation, and value.

HSV color space is a cylindrical representation in which hue is the angular component (0°–360°), saturation is the radial component (0–1), and value is the vertical component (0–1) (13). **Figure 1A** illustrates the HSV cylinder used to conceptualize the mapping strategy. **Figures 1B–D** summarize the display trajectories for grayscale, single-colorized, and multi-colorized modes, respectively.

All image processing was performed in MATLAB (MathWorks, Natick, MA, USA). Grayscale ultrasound images were normalized to an 8-bit intensity scale (0–255). Tint maps (pseudo-color maps) were generated by mapping normalized grayscale values into HSV-based color outputs and then converting to RGB for display (14). Single-color coding was implemented to emulate a commonly used clinical tint-map style, whereas the multi-colorized tint map was designed to use two distinct color ranges to enhance perceptual separation around marginal transitions.

### Thresholding and reconstruction workflow

**Figure 2:**
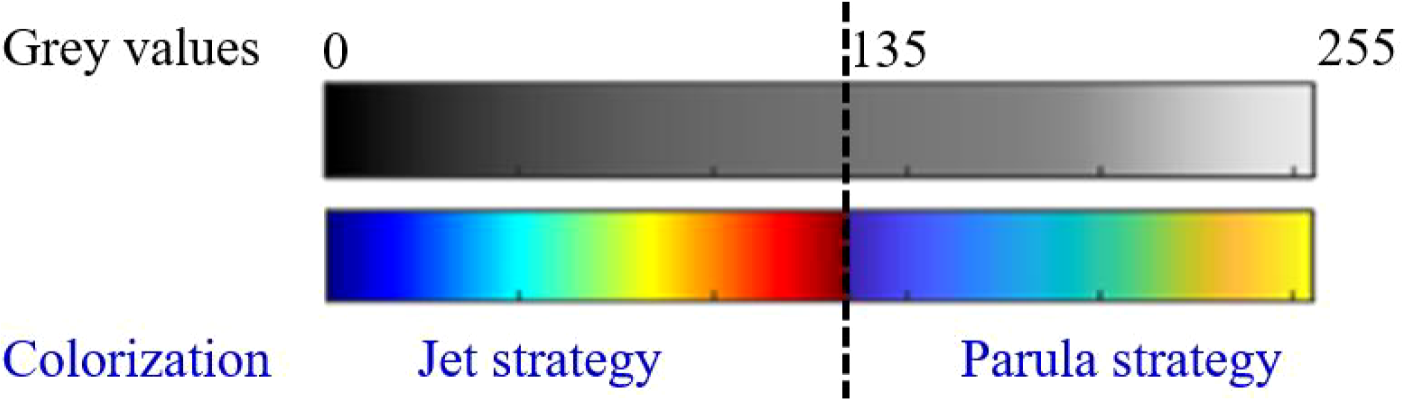
Workflow of multi-colorized tint-map reconstruction. Grayscale intensities were normalized to 0–255 and mapped using a dual-colormap strategy with a fixed threshold (T = 135), where intensities ≤T used Jet and intensities >T used Parula.

After normalization to 0–255, two color-coding strategies were applied using a fixed threshold T. Intensities ≤T were mapped using the Jet colormap, whereas intensities >T were mapped using the Parula colormap. With this dual mapping, intensities straddling the threshold are rendered with high chromatic contrast, aiming to make gradual echogenic transitions at lesion margins more visually salient than in grayscale or single-color mapping. The reconstruction scheme is summarized in **Figure 2**.

### Threshold selection

Fixed engineering choice (no optimization): The threshold was set to T = 135 on the normalized 0–255 scale as a fixed engineering parameter to place the transition near mid-range intensities and to avoid case-by-case tuning; the same threshold was applied uniformly to all cases to maximize reproducibility.

To evaluate dependence on a single threshold, we performed a robustness check by varying T within a narrow range around 135 and confirming that the qualitative presence of the marginal “halo” pattern and the primary diagnostic conclusions remained stable (details provided in the **Supplementary Table S1**).

### Reader study: identification and comparison

Ultrasound images were reviewed in three display modes (grayscale, single-colorized tint map, and multi-colorized tint map) by two radiologists with expertise in breast ultrasound. To minimize interpretation bias, given that the colorized images were derived from grayscale images, the three modes were evaluated in separate reading sessions. The order of cases within each session was randomized by an investigator not involved in image interpretation. All images were anonymized by removing patient identifiers, and readers were blinded to clinical information and reference diagnosis.

To further reduce potential recall and cross-referencing, a washout interval was implemented between reading sessions, and side-by-side comparison across modes for the same case was not permitted during interpretation. For each case and each display mode, each reader assigned one of three labels (TNBC, cyst, or fibroadenoma). Reader decisions were recorded independently using a predefined case report form.

Disagreements between the two readers were documented. A consensus discussion was then conducted to generate a consensus label for secondary analyses (e.g., illustrative examples), while primary diagnostic performance and inter-reader agreement were assessed based on the independent reads.

### Statistical analysis

Diagnostic performance was evaluated for differentiating TNBC from benign lesions (cysts and fibroadenomas). Sensitivity and specificity were calculated for each display mode. Paired comparisons between modes (grayscale *vs*. single-colorized, grayscale *vs*. multi-colorized, and single-colorized *vs*. multi-colorized) were performed using McNemar’s tests. Inter-reader agreement for each mode was quantified using Cohen’s kappa (κ) with 95% confidence intervals. A two-sided p-value <0.05 was considered statistically significant. Analyses were performed using SPSS version 22.0 for Windows (SPSS Inc., Chicago, IL, USA).

## Results

### Image reconstruction

**Figure 3:**
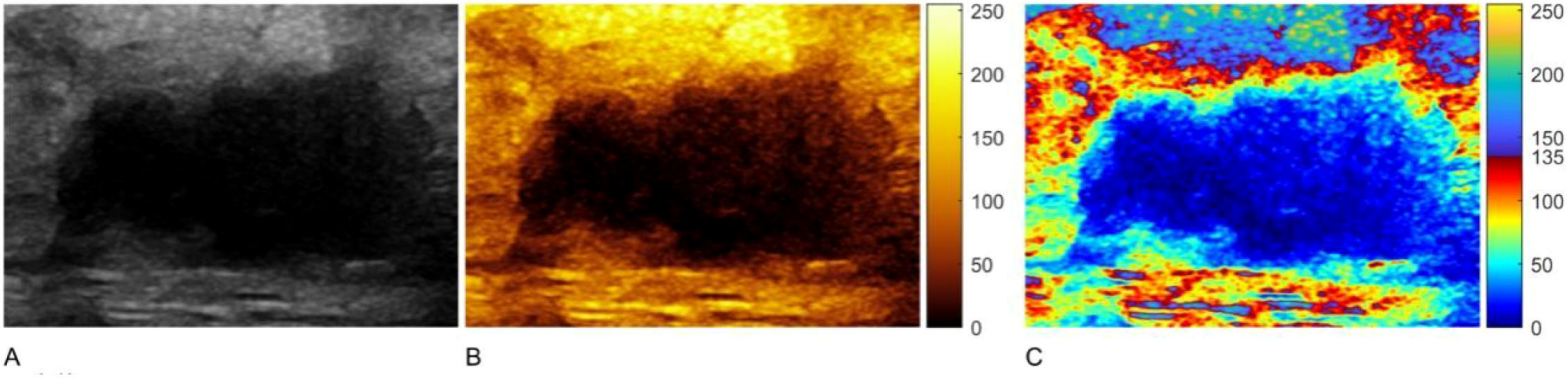
Representative TNBC lesion displayed in grayscale **(A)**, single-colorized tint map **(B)**, and multi-colorized tint map **(C)**. The multi-colorized mode applies a dual-colormap strategy with a fixed threshold (T = 135), increasing chromatic separation around marginal intensity transitions. TNBC, triple-negative breast cancer.

As illustrated in **Figure 3**, grayscale ultrasound images were converted into single-colorized and multi-colorized HSV tint-map displays. Relative to grayscale, the tint-map visualization made the intensity distribution across the lesion and adjacent background more visually separable. The multi-colorized mode applied a dual-colormap scheme split by a fixed threshold (T = 135 on the 0–255 normalized scale), which increased chromatic contrast around marginal intensity transitions and made boundary-related intensity gradients more visually conspicuous than either grayscale or single-colorized mapping.

### Imaging features on multi-colorized tint maps

**Figure 4:**
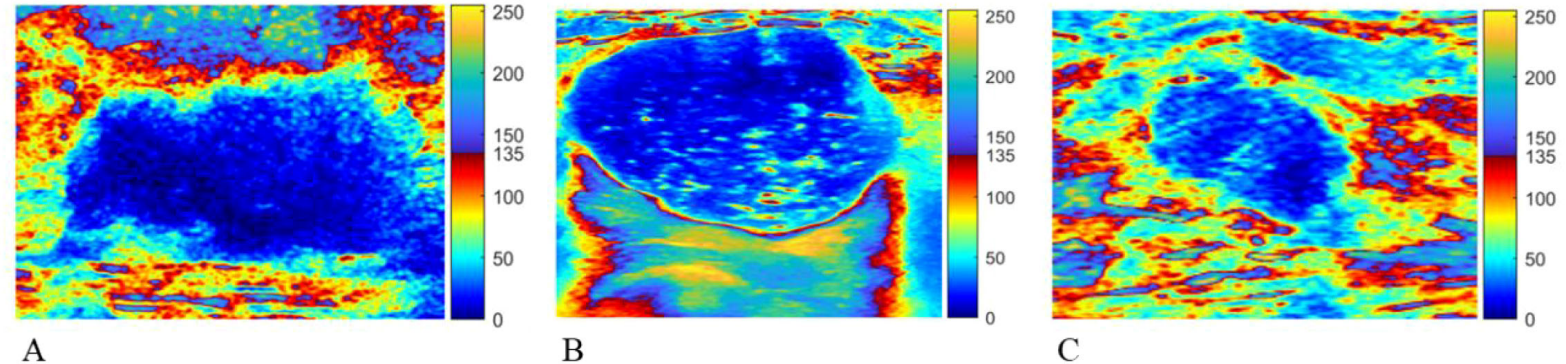
Multi-colorized tint-map examples of TNBC **(A)**, cyst **(B)**, and fibroadenoma **(C)**. A peripheral light-colored “halo” band is visible along the TNBC margin but is absent or minimal in the benign examples. TNBC, triple-negative breast cancer.

Multi-colorized tint maps facilitated the visual assessment of internal echogenicity and posterior acoustic enhancement (**Figure 4**). Cysts tended to show a visually uniform internal region and a prominent posterior acoustic enhancement that appeared relatively regular in shape, consistent with a fluid-filled lesion. Fibroadenomas and TNBCs more often exhibited a uniform-to-mottled internal appearance and less prominent posterior enhancement.

Multi-colorization also increased the perceptual salience of marginal transitions. A peripheral “halo” pattern—operationally defined as a light-colored marginal band immediately adjacent to the lesion boundary on the multi-colorized display—was frequently observed in TNBC, but was absent in cysts and uncommon in fibroadenomas (**Table 1**, **Figure 4**). Representative examples across lesion types are provided in the **Supplementary Material**.

**Table 1:** Image features of breast lesions under the multi-colorized mode.

Lesion Type	Internal echogenicity	Margin	Posterior acoustic enhancement
	Uniform/mottled	With “halo”/Without “halo”	In a regular shape/less or none
TNBC	5/11	15/1	2/14
Cyst	10/6	0/16	16/0
Fibroadenoma	2/14	3/13	3/13

Internal echogenicity: uniform/mottled; Margin: with “halo”/without “halo”; Posterior acoustic enhancement: regular-shaped enhancement/less or none. The TNBC “halo” reflects gradual grayscale transitions at the margin. The TNBC “halo” corresponds to a gradual grayscale transition at the margin.

TNBC, triple-negative breast cancer.

As summarized in **Table 1**, most TNBCs exhibited a halo pattern on the multi-colorized display, whereas only a small number of fibroadenomas showed a narrower halo-like band. Visually, the halo band was often similar in hue to the internal lesion color (e.g., a light-blue band adjacent to a darker-blue interior), consistent with a gradual rather than abrupt grayscale intensity transition from the lesion interior to surrounding tissue. This type of boundary gradient can be difficult to appreciate reliably on grayscale images alone.

**Figure 5:**
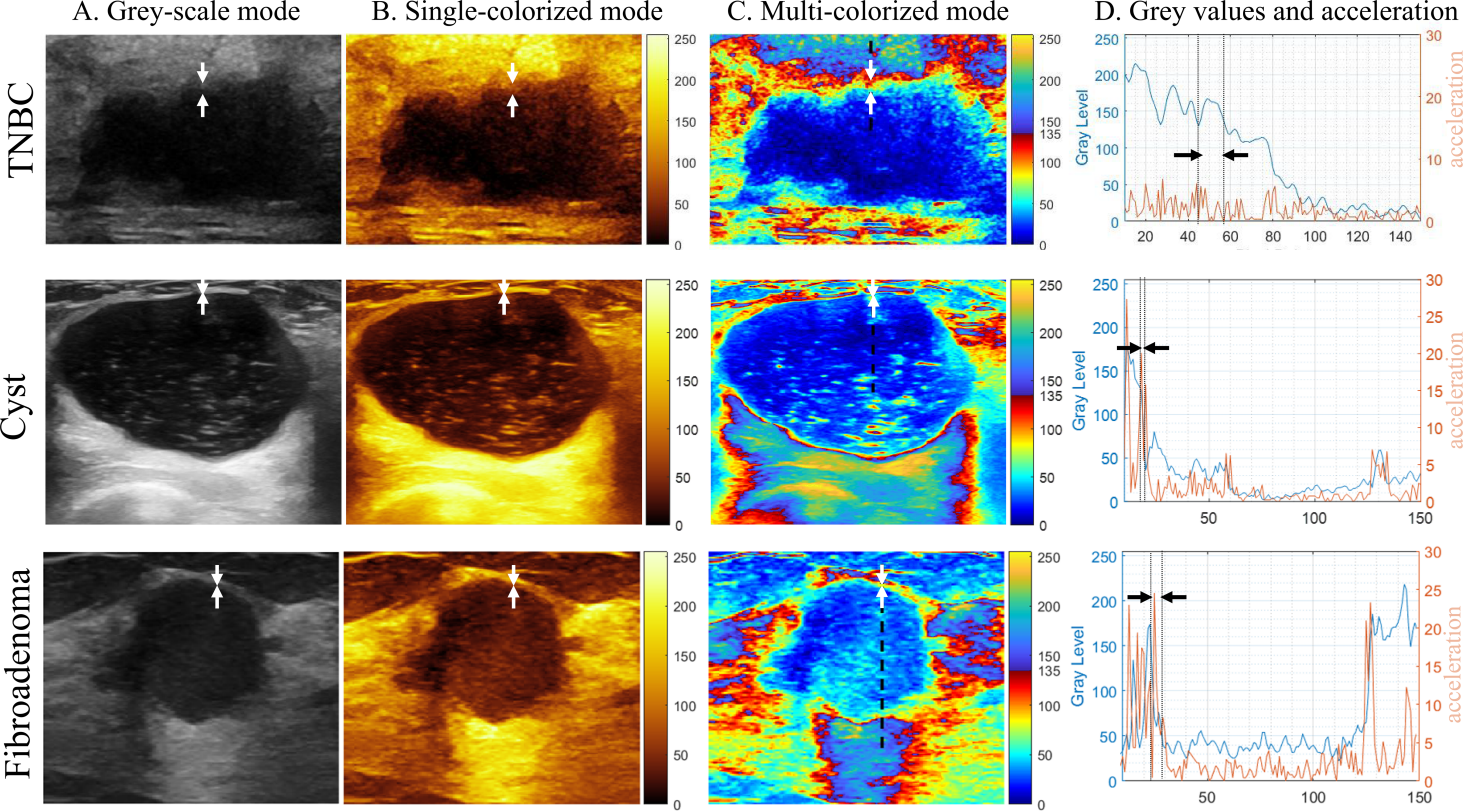
Representative TNBC, cyst, and fibroadenoma displayed in grayscale **(A)**, single-colorized **(B)**, and multi-colorized **(C)** modes. Grayscale values sampled along the dashed line in **(C)** are plotted in **(D)** (blue). The discrete second derivative (“acceleration”) of grayscale values is plotted in **(D)** (orange). The arrow-marked region indicates the lesion margin where the halo is observed on the multi-colorized display. TNBC, triple-negative breast cancer.

To illustrate the underlying grayscale behavior, a representative margin segment for TNBC, cyst, and fibroadenoma was selected. As shown in **Figure 5**, the arrow-marked region in the grayscale (**Figure 5A**) and single-colorized (**Figure 5B**) modes indicates the lesion margin where the halo is observed on the multi-colorized mode (**Figure 5C**). Grayscale intensity values sampled along the segment (dashed line in **Figure 5C**) were extracted and plotted (**Figure 5D**, blue curve). The discrete second derivative of grayscale intensity along the same segment (**Figure 5D**, orange curve) was computed as an index of how abruptly intensity changes at the boundary (i.e., boundary “sharpness”). The acceleration magnitude at the margin was <5 for TNBC, compared with 5–25 for cyst and 5–25 for fibroadenoma, indicating that TNBC showed the slowest intensity transition among the three lesion types in this illustrative analysis.

### Diagnostic performance across display modes

Both grayscale and multi-colorized modes allowed the assessment of internal echogenicity and posterior acoustic enhancement. However, multi-colorization increased the visibility of the TNBC-associated marginal halo feature, which was not readily identifiable on grayscale imaging.

For differentiating TNBC from benign lesions (cysts and fibroadenomas), sensitivity and specificity improved from 75.0% and 68.8% on grayscale ultrasound to 87.5% and 90.6% with multi-colorized tint maps, respectively (**Tables 2**, **3**).

**Table 2:** Confusion matrices for differentiating TNBC from benign lesions (n = 48).

Mode	TP (TNBC → TNBC)	FN (TNBC → Benign)	TN (Benign → Benign)	FP (Benign → TNBC)
Grayscale	12	4	22	10
Multi-colorized	14	2	29	3

TNBC, triple-negative breast cancer; TP, True Positive; FN, False Negative; TN, True Negative; FP, False Positive.

**Table 3:** Diagnostic performance summary.

Mode	Sensitivity % (TP/16)	Specificity % (TN/32)
Grayscale	75.0 (12/16)	68.8 (22/32)
Multi-colorized	87.5 (14/16)	90.6 (29/32)

TP, True Positive; TN, True Negative.

Because the same lesions were evaluated under both display modes, McNemar’s tests were performed using paired outcomes. The paired 2 × 2 tables for benign lesions (specificity comparison) and TNBC lesions (sensitivity comparison) are shown in **Tables 4A** and **4B**. Specificity was significantly higher with multi-colorization than grayscale (p = 0.046), whereas the sensitivity difference was not statistically significant (p = 0.48).

**Table 4A:** Paired outcomes for benign lesions (n = 32).

Category	Multicolorized correct	Multicolorized incorrect	Total
Grayscale correct	21	1	22
Grayscale incorrect	8	2	10
Total	29	3	32

“Correct” = classified as benign; “Incorrect” = misclassified as TNBC.

TNBC, triple-negative breast cancer.

**Table 4B:** Paired outcomes for TNBC lesions (n = 16).

Category	Multicolorized correct	Multicolorized incorrect	Total
Grayscale correct	12	0	12
Grayscale incorrect	2	2	4
Total	14	2	16

“Correct” = classified as TNBC; “Incorrect” = misclassified as benign.

TNBC, triple-negative breast cancer.

Inter-reader agreement was assessed using Cohen’s kappa (κ). Agreement was substantial for grayscale interpretation and was higher for multi-colorized interpretation (**Table 5**).

**Table 5:** Inter-reader agreement (Cohen’s κ).

Mode	κ	95% CI
Grayscale	0.76	0.60–0.92
Multi-colorized	0.88	0.76–0.99

Finally, to assess whether conclusions depended on a single threshold value, threshold robustness testing was conducted by varying the threshold around 135. Across thresholds 125–145, the marginal halo feature in TNBC remained consistently observable, and the overall diagnostic conclusions were stable (**Supplementary Table S1**).

## Discussion

As one of the most aggressive breast cancer subtypes, TNBC accounts for approximately 15%–20% of cases and is disproportionately associated with breast cancer-related mortality (2, 18, 19). A persistent clinical challenge is that TNBC may present with deceptively benign-appearing ultrasound phenotypes—round or oval shape, circumscribed margin, parallel orientation, and limited vascularity—which increases the risk of misinterpretation during routine assessment (8, 20). In this setting, incremental improvements in how diagnostically relevant boundary information is perceived on standard B-mode images are clinically meaningful, particularly when preoperative decisions depend on timely differentiation between malignancy and common benign mimickers.

Early recognition of TNBC can facilitate timely systemic therapy and may increase opportunities for breast-conserving approaches in appropriate candidates (4–6, 21). Although mammography, ultrasound, and MRI are routinely used for breast lesion evaluation, each modality presents interpretive limitations for TNBC in specific scenarios. On mammography, a substantial proportion of TNBCs may appear oval or round, which can reduce specificity in certain presentations (22, 23). MRI often shows rim enhancement in TNBC, but overlap with benign entities (e.g., internal septal enhancement in fibroadenoma) and higher cost/time burdens constrain its routine use (24, 25). Ultrasound remains a first-line modality because of accessibility and workflow compatibility, yet TNBC can mimic complicated cysts or fibroadenomas on grayscale imaging due to extreme hypoechogenicity and posterior acoustic enhancement (8). These realities motivate a practical approach that enhances perceptual salience without requiring new hardware or specialized acquisition.

From a perceptual standpoint, color-space visualization can exploit differences that are not readily captured by brightness discrimination alone (12, 26). Tint maps (pseudo-color ultrasound) assign colors to echo intensities to enhance interpretability (14). Conventional tint maps typically rely on single-hue palettes. In contrast, we explored an HSV-based multi-colorized strategy designed to increase chromatic separation specifically around marginal intensity transitions. This approach aligns with broader interest in richer image representations for breast cancer assessment, including emerging imaging and computational strategies that go beyond standard grayscale patterns (27–29). Importantly, the proposed technique functions as a transparent post-processing visualization step applied to standard B-mode images. As such, it may offer a lower barrier to translation than acquisition-dependent technologies while remaining interpretable to clinicians.

A key observation in this study was a peripheral light-colored “halo” pattern at TNBC margins on multi-colorized tint maps, which was rarely observed in cysts and uncommon in fibroadenomas. The illustrative intensity profiling along selected boundary segments further suggested that this halo corresponds to a gradual grayscale transition at the margin (i.e., a lower boundary “sharpness” as reflected by the discrete second derivative), which was not easily appreciated on grayscale images alone. This “multi-colorized halo” should be distinguished from the classic hyperechoic halo described on grayscale ultrasound, which has been reported as a malignancy-associated sign in some contexts and has been linked to receptor expression patterns in prior work (30, 31). TNBC often lacks conventional grayscale malignant signs, including a typical hyperechoic halo, and can show circumscribed margins that contribute to a benign-appearing interpretation (8). In our data, the colorized halo is best interpreted as a visualization of a boundary intensity gradient rather than direct evidence of a specific histopathologic mechanism. Nevertheless, it is biologically plausible that such gradual peripheral transitions could reflect peritumoral characteristics (e.g., cellularity gradients, stromal response, edema, or tumor–host interface heterogeneity). We therefore treat this as a testable hypothesis rather than a definitive explanation. Prospective studies correlating the halo with histopathology (e.g., tumor-edge cellularity, stromal collagen content, immune infiltration, or microvascular density) would substantially strengthen the scientific value of this observation.

From a diagnostic standpoint, multi-colorized interpretation was associated with improved specificity and overall discrimination of TNBC from benign lesions in this cohort (Results; **Tables 2**-**4**). The observation that a subset of fibroadenomas exhibited narrower halo-like patterns remains clinically important, as it indicates residual overlap even after visualization enhancement. Prior approaches to mitigate TNBC–benign confusion include optimizing ultrasound settings (e.g., reassessing tissue harmonic imaging when it obscures subtle texture cues) and leveraging computational texture-based features (8, 27). Additional ultrasound techniques may provide complementary information. Doppler and contrast-enhanced ultrasound can characterize vascularity, although TNBC may show limited blood supply in some cases (28). Elastography adds stiffness information and has been reported to improve discrimination of small or round TNBC from fibroadenoma, albeit with variable specificity across settings (29). In this context, multi-colorized tint maps may be most appropriately viewed as a pragmatic augmentation to conventional grayscale interpretation and multiparametric assessment, rather than a standalone replacement.

Inter-reader consistency is critical for any visually interpreted cue. We therefore quantified agreement using Cohen’s κ (Results; **Table 5**), providing an explicit estimate of whether multi-colorized interpretation supports consistent judgments across readers under blinded conditions. In addition, to reduce concerns that any observed effect was driven by parameter tuning, the mapping used a fixed threshold applied uniformly across cases, and robustness testing across thresholds around the chosen value showed stable halo visibility and stable diagnostic conclusions (Results; **Supplementary Table S1**). Together, these elements support the reproducibility and transparency of the proposed visualization strategy.

This study had several limitations. First, it was retrospective and single-center, which raised the possibility of selection bias and limited generalizability. In particular, we intentionally constructed a balanced cohort of extremely hypoechoic lesions (equal numbers of TNBC, cysts, and fibroadenomas) to stress-test the most clinically confusing spectrum; this design may inflate the apparent prevalence of difficult cases relative to real-world practice and can affect performance metrics when applied to broader lesion distributions. Second, ultrasound vendors and machines implement different beamforming and post-processing pipelines, which may alter intensity distributions and thereby influence color mapping. Although images were normalized and a fixed threshold was used, vendor-related variability remains a key translational concern that requires multicenter validation across systems and acquisition settings. Third, interpretation can be workflow-dependent. While the present evaluation separated grayscale and colorized sessions to reduce cross-mode bias, prospective studies should also test clinically realistic workflows (e.g., toggling or dual-frame display) and quantify whether diagnostic gains are preserved without introducing new sources of bias. Finally, cysts were confirmed by aspiration rather than histopathology, and the pathologic correlates of the halo remain uncertain; dedicated imaging–pathology correlation is needed to clarify biological underpinnings and refine interpretive criteria.

## Conclusions

HSV-based multi-colorized tint maps increased the visual salience of marginal echogenic transitions on ultrasound and revealed a peripheral halo-like feature that was frequently observed in TNBC in this cohort. This visualization was associated with improved diagnostic performance compared with grayscale interpretation when differentiating TNBC from cysts and fibroadenomas. Future work should validate robustness across ultrasound vendors in larger, multicenter cohorts and clarify the histopathologic basis of the halo through prospective imaging–pathology correlation.

## Data Availability

The original contributions presented in the study are included in the article/**Supplementary Material**. Further inquiries can be directed to the corresponding author.

## Supplementary material

Supplementary Table 1 Threshold robustness around 135.
