# Supplementary material for: Multi-colorized tint map for distinguishing triple-negative breast cancers from cysts and fibroadenomas based on the tumor margin
Source: Front Oncol. 2026 Feb 5;16:1741453. doi: 10.3389/fonc.2026.1741453 (PMC12916380; doi:10.3389/fonc.2026.1741453)
Supplement: Supplementary file 1 [file DataSheet1.docx]

**Supplementary document**

| 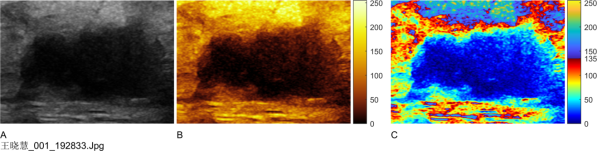  Figure S1. A 60-year-old female patient presenting with a left breast lesion. The patient incidentally discovered a breast mass 2 days ago. The mass has a very low echogenicity. A core needle biopsy was performed, and pathological results confirmed a diagnosis of triple-negative breast cancer (TNBC). A, greyscale mode; B, single-colorized mode; C, multi-colorized mode showing the mass with a “halo”. |
| --- |
| 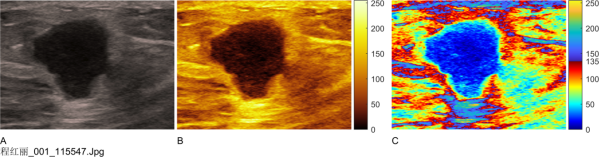  Figure S2. A 34-year-old female patient with a palpable mass in the upper outer quadrant of the right breast, detected during routine self-examination. The patient has no history of breast lesion but has a sister with breast cancer. The ultrasonography revealed a spiculated mass with extreme hypo-echogenicity, and the subsequent biopsy indicated the invasive ductal carcinoma. Tumor markers showed triple-negative status, guiding the treatment approach. A, greyscale mode; B, single-colorized mode; C, multi-colorized mode showing the mass with a “halo”. |
| 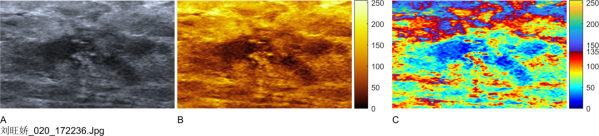  Figure S3. A 32-year-old female patient presenting with a right breast mass. The patient noted a firm lump that had been increasing in size over the past 4 months, accompanied by the occasional stabbing pain. The ultrasonography revealed the irregular margin and hypo-echogenicity. A core needle biopsy was performed, and the histopathological analysis confirmed TNBC. A, greyscale mode; B, single-colorized mode; C, multi-colorized mode showing the mass with a “halo”. |
| 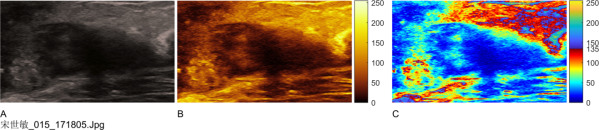  Figure S4. A 54-year-old female with a history of treated left breast carcinoma, now presenting with a new mass in the contralateral breast. The ultrasonography detected an extremely hypoechoic mass. Stereotactic biopsy confirmed invasive lobular carcinoma. Molecular testing was performed, indicating estrogen receptor-negative, HER2-negative status. A, greyscale mode; B, single-colorized mode; C, multi-colorized mode showing the mass with a “halo”. |
| 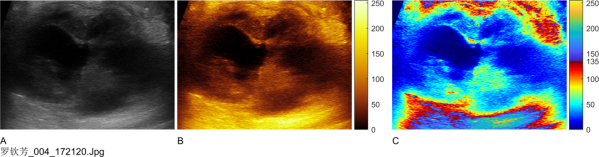  Figure S5. A 55-year-old woman with the new onset breast pain and a palpable mass in the right breast. The past ultrasonography suggested a suspicious mass with the benign fibrocystic changes. The current ultrasonography revealed a complex cystic and solid mass, and the fine needle aspiration was conducted. Cytology showed atypical cells, prompting a core biopsy, which confirmed a diagnosis of invasive lobular carcinoma. Hormone receptor testing showed estrogen receptor-negative, HER2-negative status. A, greyscale mode; B, single-colorized mode; C, multi-colorized mode showing the mass with a “halo”. |
| 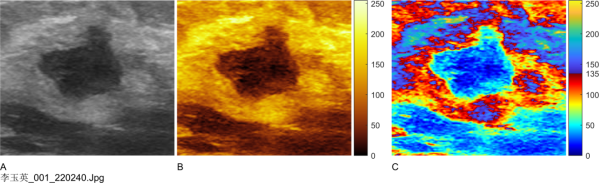  Figure S6. A 46-year-old female patient presenting with a right breast lesion. The patient identified a breast lump one month ago. The previous mammogram was normal, and there is a family history of breast cancer in her mother. A core needle biopsy was performed, and the histopathological examination revealed it was a TNBC. A, greyscale mode; B, single-colorized mode; C, multi-colorized mode showing the mass with a “halo”. |
| 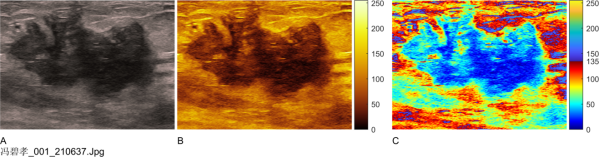  Figure S7. A 50-year-old woman presenting with a palpable, non-tender mass in the left breast. The patient has a family history of breast cancer. The ultrasonography confirmed a hypoechoic mass. Core biopsy results indicated an invasive ductal carcinoma with estrogen receptor negative and HER2-negative status. A, greyscale mode; B, single-colorized mode; C, multi-colorized mode showing the mass with a “halo”. |
| 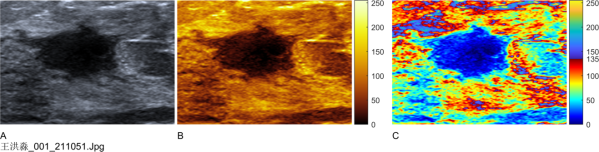  Figure S8. A 64-year-old woman with a palpable mass in the left breast, detected during the self-examination. The patient reported an occasional pain in the affected area. The ultrasonography suggested an irregular mass with the extreme hypo-echogenicity. A subsequent biopsy confirmed an invasive breast cancer, with negative findings for estrogen receptor, progesterone receptor, and no HER2 amplification. A, greyscale mode; B, single-colorized mode; C, multi-colorized mode showing the mass with a “halo”. |
| 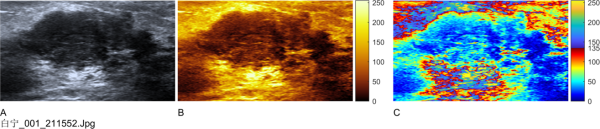  Figure S9. A 52-year-old female patient presenting with a left breast mass. The patient noted a firm, non-tender lump that had been increasing in size over the past 6 months. She has a personal history of the right breast cancer 8 years prior, treated with the lumpectomy and radiation. A core needle biopsy was performed, and the histopathological analysis confirmed TNBC. A, greyscale mode; B, single-colorized mode; C, multi-colorized mode showing the mass with a “halo”. |
| 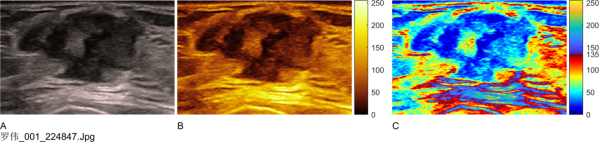  Figure S10. A 42-year-old female presenting with the dimpling and thickening skin of the left breast over the past 3 months. The clinical examination revealed a firm mass with the associated peau d’orange appearance. The ultrasonography suggested a cystic and solid mass A biopsy was performed, confirming TNBC. A, greyscale mode; B, single-colorized mode; C, multi-colorized mode showing the mass with a “halo”. |
| 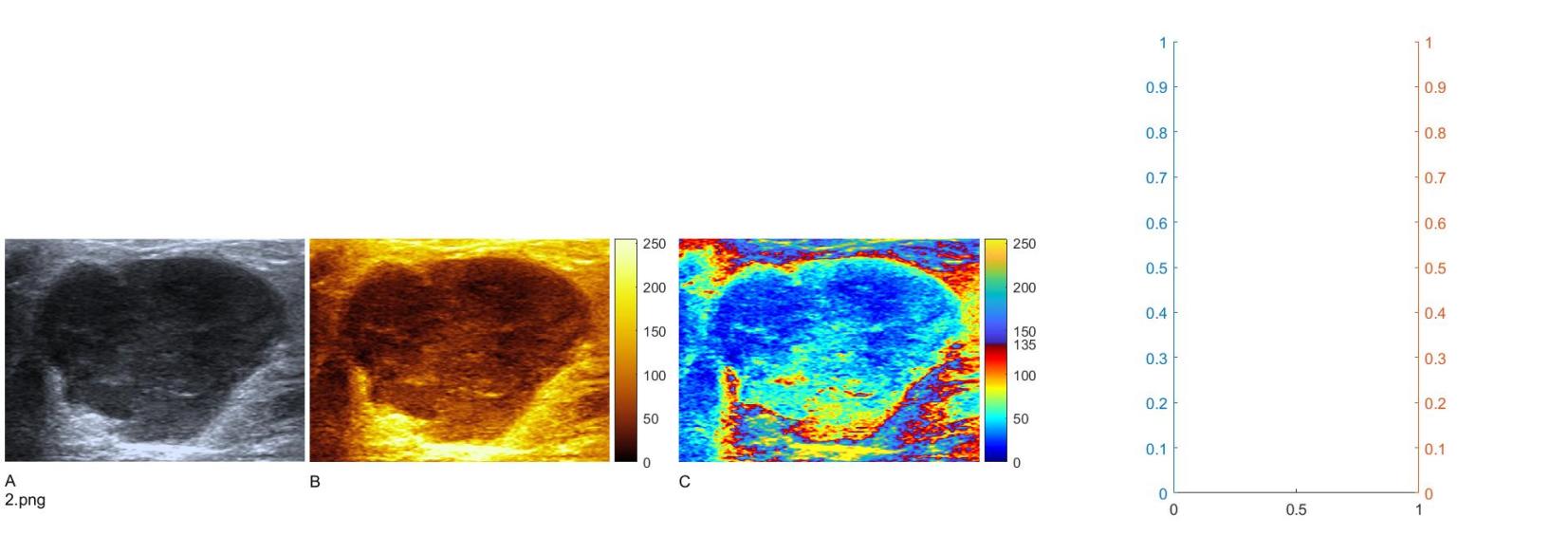  Figure S11. A 59-year-old woman with a history of bilateral benign breast cysts, now presenting with a new mass in the right breast. The ultrasonography revealed an extremely hypoechoic mass, and the core biopsy showed invasive papillary carcinoma. Hormone receptor testing indicated estrogen and progesterone receptor negativity. A, greyscale mode; B, single-colorized mode; C, multi-colorized mode showing the mass with a “halo”. |
| 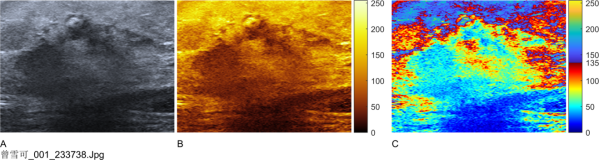  Figure S12. A 47-year-old female presenting with a rapidly enlarging mass in the right breast over the past 2 months. She reported no pain, but noted redness and warmth in the affected area. Mammography and MRI revealed a large, irregular mass with the increased vascularity. The ultrasonography showed a hypoechoic mass with the irregular and blurred margin. The core biopsy confirmed invasive ductal carcinoma with the lympho-vascular invasion. A, greyscale mode; B, single-colorized mode; C, multi-colorized mode showing the mass without a “halo”. |
| 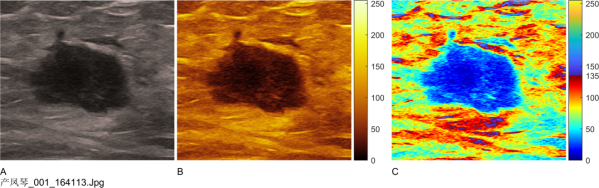  Figure S13. A 48-year-old female with a family history of the breast and ovarian cancer, presenting with a palpable lump in the upper inner quadrant of the left breast. The ultrasonography showed a spiculated, hypoechoic mass. Core biopsy confirmed triple-negative invasive ductal carcinoma. A, greyscale mode; B, single-colorized mode; C, multi-colorized mode showing the mass with a “halo”. |
| 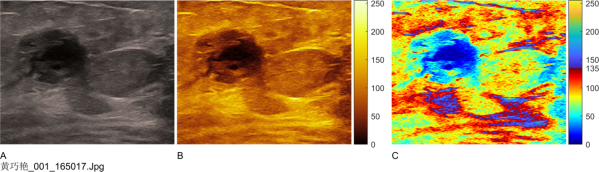  Figure S14. A 45-year-old female with a palpable right breast mass. The patient reported the gradual increasing size of the lump over the past 6 months without any pain. The ultrasonography suggested a cystic and solid mass. A fine needle aspiration was conducted, and the cytological analysis confirmed TNBC. A, greyscale mode; B, single-colorized mode; C, multi-colorized mode showing the mass with a “halo”. |
| 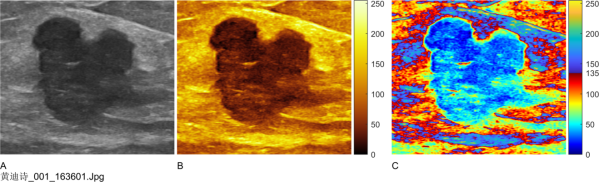  Figure S15. A 50-year-old woman presenting with a painless lump in the right breast, discovered 3 weeks ago. On palpation, the mass was found to be hard and mobile. The ultrasonography revealed an extremely hypo-echoic mass with the irregular margin A core biopsy confirmed invasive carcinoma, and the tumor was found to be HER2-negative. A, greyscale mode; B, single-colorized mode; C, multi-colorized mode showing the mass with a “halo”. |
| 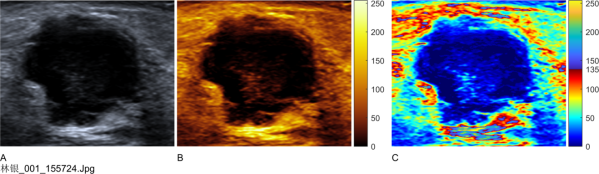  Figure S16. A 47-year-old female with a newly discovered breast mass in the left breast. The patient reported no pain but noticed the mass had enlarged over the past 4 months. The ultrasonography revealed a mass with the extreme hypo-echogenicity and irregular margin. Biopsy confirmed TNBC with a high Ki-67 index, suggesting a more aggressive tumor. A, greyscale mode; B, single-colorized mode; C, multi-colorized mode showing the mass with a “halo”. |
| 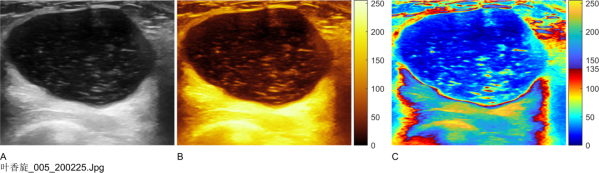  Figure S17. A 28-years-old female patient with a breast mass over ten years, gradually increasing in size. The ultrasonography suggested a mass with the extremely hypo-echogenicity and oval margin. The patient underwent aspiration and the cyst sclerotherapy. A, greyscale mode; B, single-colorized mode; C, multi-colorized mode showing the mass with a regular shape of posterior acoustic enhancement (PAE) and no “halo”. |
| 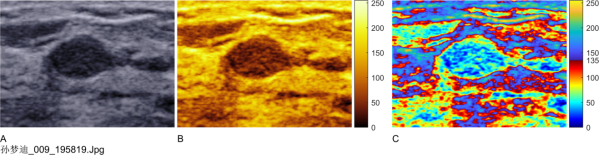  Figure S18. A 36-year-old woman with a breast mass detected by the routine mammography. Further ultrasound evaluation revealed an extremely hypoechoic mass with the possibility of a cyst. Under the ultrasound guidance, the clear fluid was aspirated. A, greyscale mode; B, single-colorized mode; C, multi-colorized mode showing the mass without a PAE or a “halo”. |
| 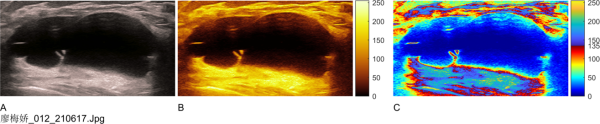  Figure S19. A 39-year-old female with a painful lump in the left breast, which had enlarged over the past 6 months. The ultrasonography considered a cyst with some internal septations. The cyst was confirmed by the aspiration. A, greyscale mode; B, single-colorized mode; C, multi-colorized mode showing the mass with a regular shape of PAE and no “halo”. |
| 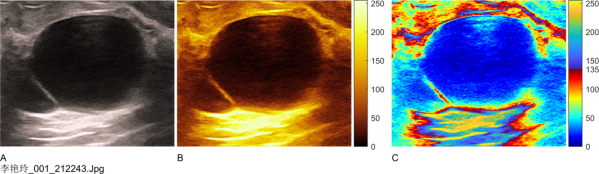  Figure S20. A 45-year-old female patient presenting with a palpable lump in the left breast, gradually enlarging over the past 12 months. The patient has no significant family history of breast cancer. The ultrasonography considered a cyst with some internal septations. The cyst was confirmed by the aspiration. A, greyscale mode; B, single-colorized mode; C, multi-colorized mode showing the mass with a regular shape of PAE and no “halo”. |
| 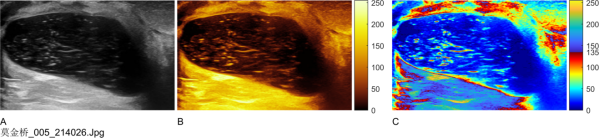  Figure S21. A 46-year-old woman with a progressively enlarging lump in the left breast. The ultrasonography considered a cyst with the poor sound transmission. The cyst was confirmed by the aspiration. A, greyscale mode; B, single-colorized mode; C, multi-colorized mode showing the mass with a regular shape of PAE and no “halo”. |
| 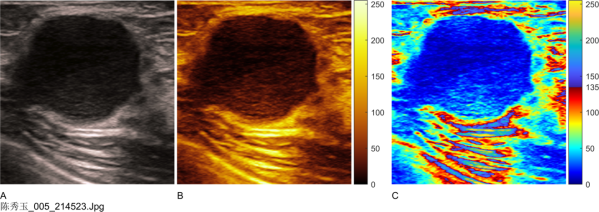  Figure S22. A 36-year-old woman with a breast mass detected by the routine screening mammography. The patient has a history of fibrocystic breast changes and reported the intermittent pain. The ultrasonography revealed an extremely hypo-echoic mass and an irregular margin. Contrast-enhanced ultrasound (CEUS) suggested its cystic nature and the aspiration confirmed it. A, greyscale mode; B, single-colorized mode; C, multi-colorized mode showing the mass with a regular shape of PAE and no “halo”. |
| 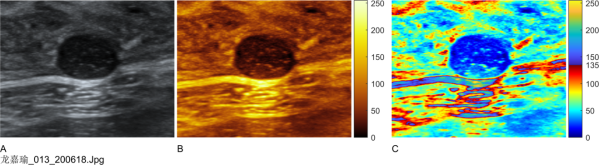  Figure S23. A 27-year-old woman with a breast mass detected by the routine screening mammography. The ultrasonography revealed an extremely hypo-echoic mass and an oval margin. Contrast-enhanced ultrasound (CEUS) suggested its cystic nature and the aspiration confirmed it. A, greyscale mode; B, single-colorized mode; C, multi-colorized mode showing the mass with a regular shape of PAE and no “halo”. |
| 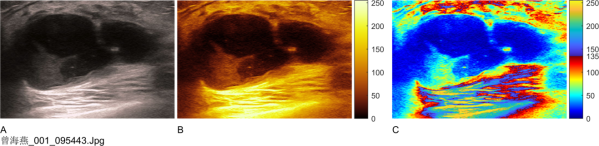  Figure S24. A 50-year-old female with a breast lump that has slowly increased in size over the past five years. Mammography revealed an area of low density. The ultrasonography considered a cystic and solid mass with the septation. Contrast-enhanced ultrasound (CEUS) suggested its cystic nature and the aspiration confirmed it. A, greyscale mode; B, single-colorized mode; C, multi-colorized mode showing the mass with a regular shape of PAE and no “halo”. |
| 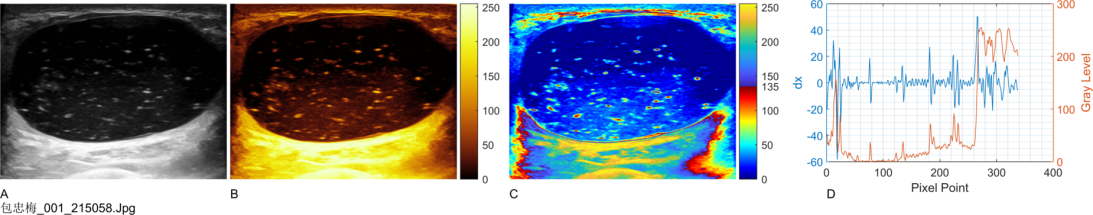Figure S25. A 35-year-old female patient presenting with a painful lump in the left breast that fluctuated in size during the menstrual cycle. The ultrasonography considered a cyst with some deposits. The cyst was confirmed by the aspiration (the cystic fluid was cloudy). A, greyscale mode; B, single-colorized mode; C, multi-colorized mode showing the mass with a regular shape of PAE and no “halo”. |
| 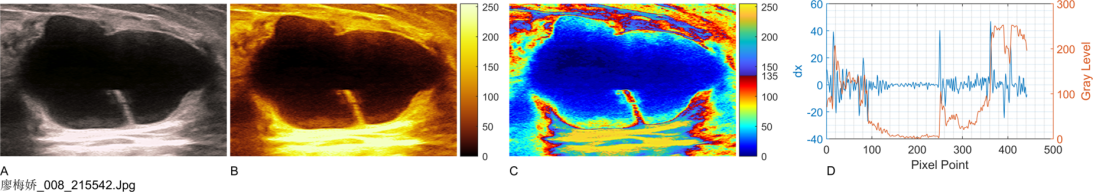  Figure S26. A 42-year-old female patient presenting with a palpable lump in the right breast. The ultrasonography considered a cyst with some internal septations. The cyst was confirmed by the aspiration. A, greyscale mode; B, single-colorized mode; C, multi-colorized mode showing the mass with a regular shape of PAE and no “halo”. |
| 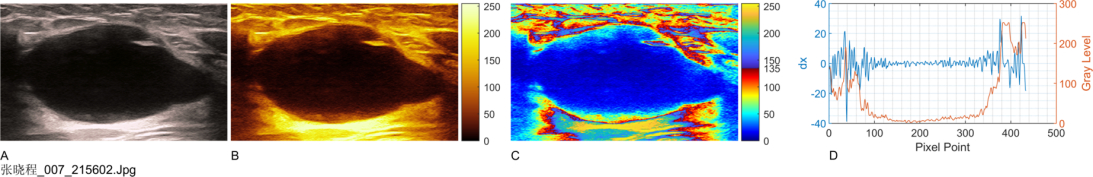Figure S27. A 50-year-old female patient presenting with a palpable mass in the upper outer quadrant of the right breast. The ultrasonography considered a cyst. The cyst was confirmed by the aspiration. A, greyscale mode; B, single-colorized mode; C, multi-colorized mode showing the mass with a regular shape of PAE and no “halo”. |
| 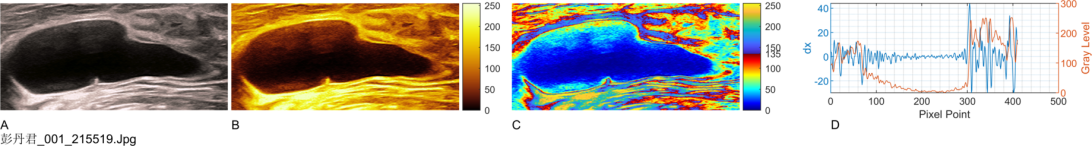  Figure S28. A 45-year-old woman noticed a non-painful lump in her left breast. The ultrasonography considered a cyst. The cyst was confirmed by the aspiration. A, greyscale mode; B, single-colorized mode; C, multi-colorized mode showing the mass with a regular shape of PAE and no “halo”. |
| 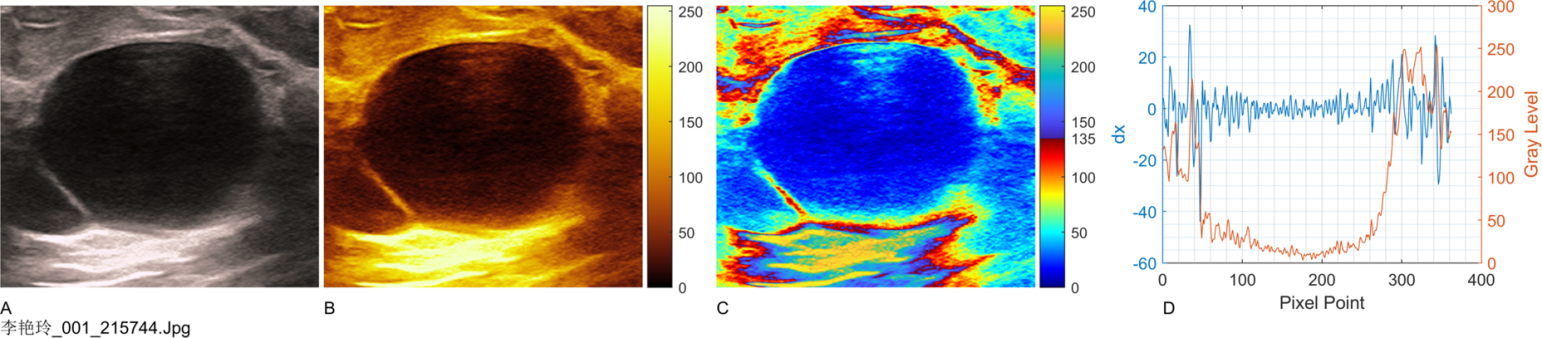Figure S29. A 48-year-old female patient with a new, firm lump in the right breast that had been present for approximately three months. The lump was mildly tender, and the patient was concerned about it increasing in size. The ultrasonography considered a cyst with some septations. The cyst was confirmed by the aspiration. A, greyscale mode; B, single-colorized mode; C, multi-colorized mode showing the mass with a regular shape of PAE and no “halo”. |
| 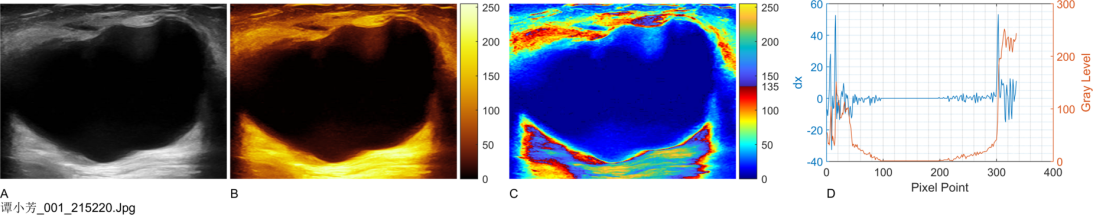  Figure S30. A 38-year-old female patient presenting with a breast lump in the right upper quadrant over approximately six weeks. The lump was described as soft, mobile, and painless. The ultrasonography considered a cyst with a diameter of 3 cm. The cyst was confirmed by the aspiration. A, greyscale mode; B, single-colorized mode; C, multi-colorized mode showing the mass with a regular shape of PAE and no “halo”. |
| 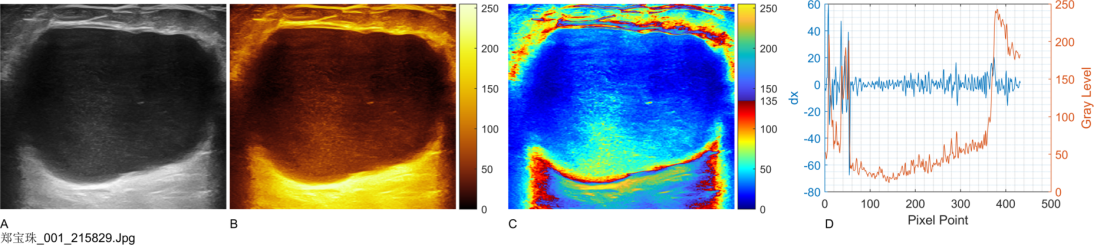Figure S31. A 45-year-old female patient presenting with a palpable mass in the right breast. The mass was described as tender, particularly before the menstruation. The ultrasonography considered a cyst with the poor sound transmission. The cyst was confirmed by the aspiration. A, greyscale mode; B, single-colorized mode; C, multi-colorized mode showing the mass with a regular shape of PAE and no “halo”. |
| 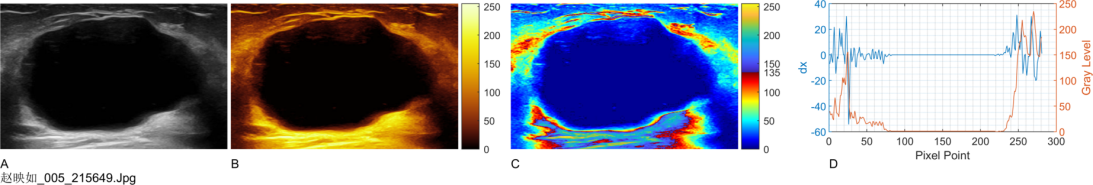  Figure S32. A 50-year-old female patient presenting with a palpable, soft, non-tender lump in the left breast. The lump had increased in size over the last two months. The ultrasonography considered a cyst. The cyst was confirmed by the following aspiration. A, greyscale mode; B, single-colorized mode; C, multi-colorized mode showing the mass with a regular shape of PAE and no “halo”. |
| 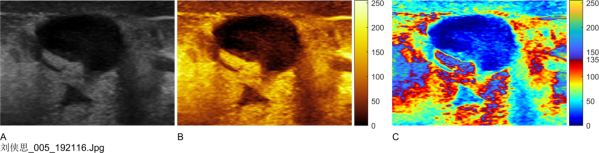  Figure S33. A 34-year-old woman with a mobile, non-tender lump in the left breast. The lump had enlarged over one year. The ultrasonography presented an extremely hypoechoic mass with a regular margin. Due to the progressive growth of the mass, the lumpectomy was recommended. The pathological result was fibroadenoma. A, greyscale mode; B, single-colorized mode; C, multi-colorized mode showing an indefinite PAE and “halo”. |
| 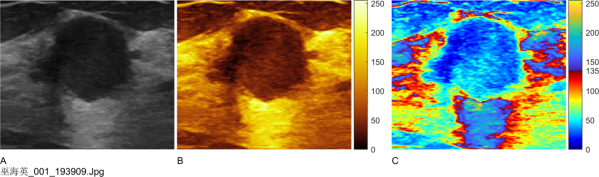  Figure S34. A 40-year-old woman presenting with a palpable mass in the right breast, noticed during the routine self-examination. The ultrasonography showed an extremely hypoechoic and well-circumscribed mass with PAE. Given the patient’s anxiety and preference for removal, the lumpectomy was performed. The pathological result was fibroadenoma with the significant mucinous degeneration. A, greyscale mode; B, single-colorized mode; C, multi-colorized mode showing a regular shape of PAE and an indefinite “halo”. |
| 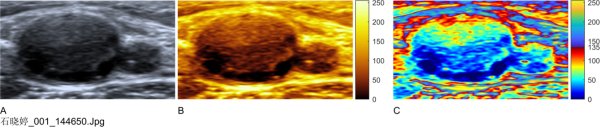  Figure S35. A 32-year-old female with a firm, mobile mass in the left breast, noticed approximately 8 months ago. The patient reported slight discomfort associated with the mass. The ultrasonography showed an extremely hypoechoic and well-circumscribed mass. Due to the persistent symptoms, the surgical excision was performed. The pathology confirmed a benign fibroadenoma. A, greyscale mode; B, single-colorized mode; C, multi-colorized mode showing no PAE or a “halo”. |
| 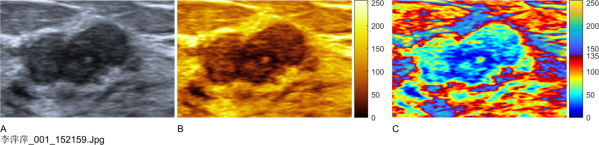  Figure S36. A 40-year-old woman was previously found to have a breast nodule on ultrasonography. The patient reported no associated symptoms. The ultrasonography showed a well circumscribed, and hypoechoic mass, but the margin is somewhat irregular. The biopsy confirmed it was a fibroadenoma. A, greyscale mode; B, single-colorized mode; C, multi-colorized mode showing no PAE or a “halo”. |
| 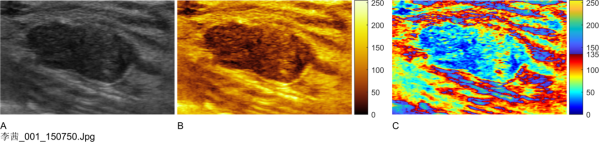  Figure S37. A 30-year-old female with a firm, mobile mass in the upper outer quadrant of the right breast, initially noticed 6 months ago. The ultrasonography revealed a homogeneous, hypoechoic mass with well-circumscribed margin, suggestive of a fibroadenoma. The biopsy confirmed the diagnosis. A, greyscale mode; B, single-colorized mode; C, multi-colorized mode showing no PAE or a “halo”. |
| 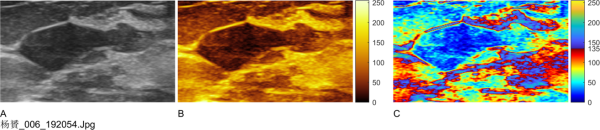  Figure S38. A 28-year-old woman with a palpable lump in the left breast, found by the routine screening. Ultrasound examination showed an extremely hypoechoic, homogeneous, and well-circumscribed lesion with somewhat irregular margin. The following biopsy demonstrated it was a fibroadenoma. A, greyscale mode; B, single-colorized mode; C, multi-colorized mode showing no PAE or “halo”. |
| 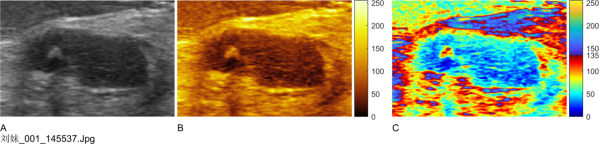  Figure S39. A 47-year-old woman with a firm lump in the upper outer quadrant of the left breast, noticed approximately 6 months ago. The ultrasonography showed a mass with the well-circumscribed margin and suspicious microcalcification. The lumpectomy was performed, and the pathology demonstrated it was a fibroadenoma. A, greyscale mode; B, single-colorized mode; C, multi-colorized mode showing no PAE or “halo”. |
| 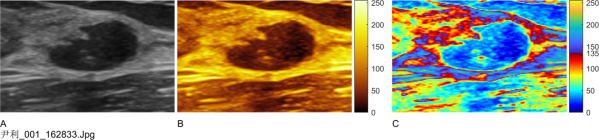  Figure S40. A 55-year-old woman with a lump in the right breast. Ultrasound examination showed an extremely hypoechoic, homogeneous, and irregular lesion. The following biopsy demonstrated it was a fibroadenoma. A, greyscale mode; B, single-colorized mode; C, multi-colorized mode showing no PAE or “halo”. |
| 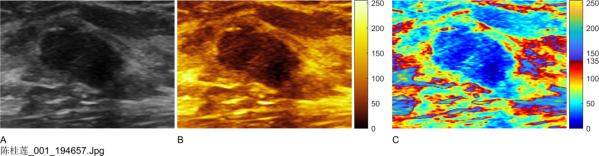  Figure S41. A 33-year-old female with a palpable lump in the left breast. The ultrasonography showed an extremely hypo-echoic mass with oval shape and non-parallel orientation. The patient underwent the biopsy, demonstrating it was a fibroadenoma. A, greyscale mode; B, single-colorized mode; C, multi-colorized mode showing no PAE or “halo”. |
| 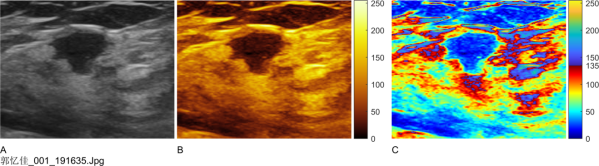  Figure S42. A 30-year-old woman presented with a lobulated, hypoechoic lesion in the left breast. Due to the non-parallel orientation, the malignancy was considered. Both the core needle biopsy and the following lumpectomy demonstrated it was a fibroadenoma. A, greyscale mode; B, single-colorized mode; C, multi-colorized mode showing no PAE or “halo”. |
| 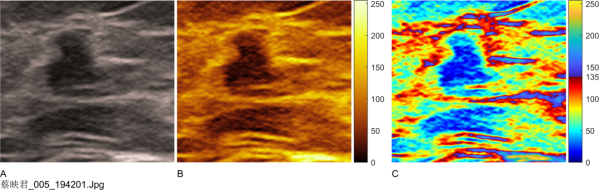  Figure S43. A 32-year-old female presented with a lobulated, hypoechoic lesion in the left breast. Due to the non-parallel orientation, the malignancy was considered. Both the core needle biopsy and the following lumpectomy demonstrated it was a fibroadenoma. A, greyscale mode; B, single-colorized mode; C, multi-colorized mode showing no PAE but an indefinite “halo”. |
| 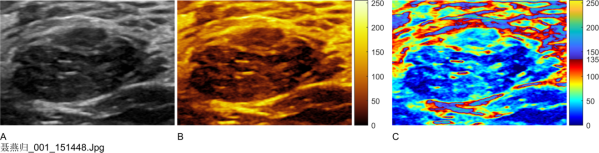  Figure S44. A 35-year-old woman with a palpable, painless lump in the left breast. The patient has no significant family history of breast disease. The ultrasonographic features including oval shapes, regular margin and the parallel orientation. Due to the anxiety, the patient required the biopsy. The pathologic result was a fibroadenoma. A, greyscale mode; B, single-colorized mode; C, multi-colorized mode showing no PAE or “halo”. |
| 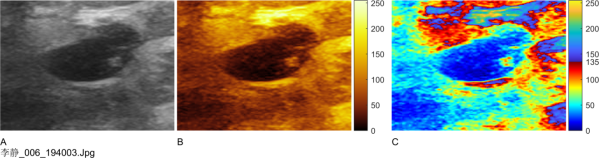  Figure S45. A 28-year-old female presented a painless mass in her right breast during the self-examination. The ultrasonography revealed a well-defined, hypoechoic lesion with the calcification. The biopsy was performed, demonstrating it was a fibroadenoma. A, greyscale mode; B, single-colorized mode; C, multi-colorized mode showing no PAE or “halo”. |
| 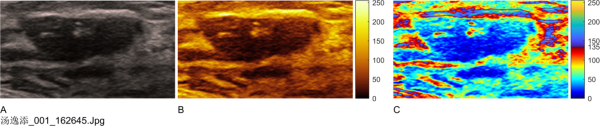  Figure S46. A 34-year-old woman presented with a firm, palpable mass in the left breast, detected by self-examination 6 months earlier. The ultrasonography indicated a solid, hypoechoic lesion with the calcification. The biopsy was performed, demonstrating it was a fibroadenoma. A, greyscale mode; B, single-colorized mode; C, multi-colorized mode showing no PAE or “halo”. |
| 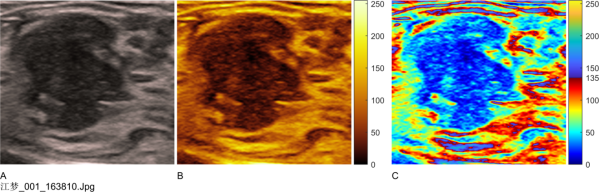  Figure S47. A 34-year-old woman with a palpable and mobile lump in the left breast over four months. The patient reported no symptoms. The ultrasonographic features including hypo-echogenicity, lobular shape and non-parallel orientation, thereby performing the biopsy. The pathologic result was a fibroadenoma. The biopsy was performed, demonstrating it was a fibroadenoma. A, greyscale mode; B, single-colorized mode; C, multi-colorized mode showing no PAE or “halo”. |
| 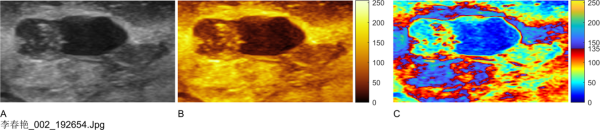  Figure S48. A 44-year-old woman with a lump in the left breast. Ultrasound examination showed an extremely hypoechoic, heterogeneous, and well-circumscribed lesion. The following biopsy demonstrated it was a fibroadenoma. A, greyscale mode; B, single-colorized mode; C, multi-colorized mode showing no PAE or “halo”. |
