# Supplementary material for: Multi-colorized tint map for distinguishing triple-negative breast cancers from cysts and fibroadenomas based on the tumor margin
Source: Front Oncol. 2026 Feb 5;16:1741453. doi: 10.3389/fonc.2026.1741453 (PMC12916380; doi:10.3389/fonc.2026.1741453)
Supplement: Supplementary Table 1 — Threshold robustness around 135. [file Table1.docx]

Supplementary Table S1. Threshold robustness around 135

| **Threshold** | **Sensitivity % (TP/16)** | **Specificity % (TN/32)** | **TNBC “halo” presence (n/16)** | **Benign “halo” presence (n/32)** |
| --- | --- | --- | --- | --- |
| 125 | 87.5 (14/16) | 87.5 (28/32) | 14 | 4 |
| 130 | 87.5 (14/16) | 90.6 (29/32) | 15 | 3 |
| 135 | 87.5 (14/16) | 90.6 (29/32) | 15 | 3 |
| 140 | 81.3 (13/16) | 93.8 (30/32) | 14 | 2 |
| 145 | 81.3 (13/16) | 90.6 (29/32) | 13 | 2 |

TNBC, triple-negative breast cancer.
